# Comparison of Fracture Identification Using Different Definitions in Healthcare Administrative (Claims) Data

**DOI:** 10.3390/pharmacy11020053

**Published:** 2023-03-14

**Authors:** Natalia Konstantelos, Andrea M. Burden, Angela M. Cheung, Sandra Kim, Paul Grootendorst, Suzanne M. Cadarette

**Affiliations:** 1 Leslie Dan Faculty of Pharmacy, University of Toronto, Toronto, ON M5S 3M2, Canada; 2 ETH Zurich, 8092 Zurich, Switzerland; 3 Dalla Lana School of Public Health, University of Toronto, Toronto, ON M5T 3M7, Canada; 4 Department of Medicine, University Health Network, University of Toronto, Toronto, ON M5S 1A8, Canada; 5 Women’s College Hospital, Toronto, ON M5S 1B2, Canada; 6 ICES, Toronto, ON M4N 1P8, Canada; 7 Eshelman School of Pharmacy, University of North Carolina, Chapel Hill, NC 27599, USA

**Keywords:** drug safety, research methodology, osteoporotic fractures, osteoporosis, pharmacoepidemiology

## Abstract

We identified inconsistency in fracture definitions in a prior review of studies that utilized claims data. Here, we aimed to compare fracture rates estimated using thirteen hip and seven radius/ulna fracture definitions. Our primary analysis compared results in a cohort of 120,363 older adults treated with oral bisphosphonates for ≥3 years. The most inclusive definition (hip: inpatient or emergency diagnosis; radius/ulna: inpatient, emergency, or outpatient diagnosis) served as a referent to compare the number and proportion of fractures captured. In sensitivity analyses, we considered a 180-day washout, excluded fractures associated with trauma; and hip only, excluded: (1) subtrochanteric fractures, and (2) hip replacement procedures. Hip fractures varied by definition in number (52–8058) and incidence (0.7–111.8/10,000 person-years). The second most inclusive definition required one inpatient diagnosis and identified 8% fewer hip fractures than the referent. Excluding hip replacements missed 33% of hip fractures relative to the primary analysis. Radius/ulna fractures also ranged in number (1589–6797) and incidence (22.0–94.3/10,000 person-years). Outpatient data were important, when restricted to inpatient or emergency data, only 78% of radius/ulna fractures were identified. Other than hip replacement procedures, sensitivity analyses had minimal impact on fracture identification. Analyses were replicated in a cohort of patients treated with long-term glucocorticoids. This study highlights the importance and impact of coding decisions on fracture outcome definitions. Further research is warranted to inform best practice in fracture outcome identification.

## 1. Introduction

Healthcare administrative (claims) data are commonly used to track fracture trends in large populations and answer clinical questions about fracture risk in the real world. Given the importance of real-world evidence in estimating drug effectiveness and safety [1,2], there have been recent calls for transparency and consistency in outcome definitions used to improve reproducibility [3]. In a recent scoping review, we identified wide variation and little transparency in fracture outcome definitions among osteoporosis drug effectiveness studies published from 2000 to 2020 that used claims data to define outcomes [4]. The lack of consistency in fracture outcome definitions across studies is concerning, especially considering the impact real-world evidence can have in clinical settings, including fracture risk factor identification and estimates of drug effects that inform clinical practice guidelines. However, to our knowledge, no studies have directly compared how these definitions perform. Therefore, we aimed to compare the different claims-based fracture definitions and evaluate the impact on fracture identification and incidence.

## 2. Materials and Methods

### 2.1. Data Sources

We leveraged an existing cohort of 120,368 long-term oral bisphosphonate users (≥3 years of continuous therapy with ≥80% adherence quantified by proportion of days covered) in Ontario, Canada aged ≥ 66 years [5,6]. We restricted inclusion in the current study to patients that met the criteria for long-term bisphosphonate cohort entry as of 1 April 2003, when ICD-10-CA coding was established in Ontario [7]. We followed patients until 31 March 2020. This study included those ≥ 66 years to ensure at least one year free from oral bisphosphonate use before cohort entry since comprehensive public drug coverage in Ontario begins at age 65.

We replicated our findings in a second cohort with different characteristics to strengthen our conclusions and consider if our results are generalizable. Our secondary cohort consisted of 203,358 chronic oral glucocorticoid users (>2 glucocorticoid dispensations of at least 450 mg prednisone equivalent over 6 months) [8,9]. We similarly restricted inclusion in the current study to community-dwelling patients with index since 1 April 2003. These cohorts were previously constructed using databases that were linked using unique encoded identifiers and analyzed at ICES.

### 2.2. Outcomes (Fracture Definitions)

We identified hip and radius/ulna fractures in each cohort, using definitions identified in our previous review of 57 papers that studied osteoporosis treatment and fracture risk using healthcare administrative data [4], supplemented with expert opinion (SMC). We specifically considered hip and radius/ulna fractures for two reasons. First, from a clinical perspective, hip and radius/ulna fractures are among the most common fracture sites included in osteoporosis drug effects studies [4]. Second, from a methodological perspective, we aimed to compare differences in the data sources required to identify fracture sites due to the differences in the clinical treatment of these fractures, as hip fractures are typically treated in hospital, and radius/ulna fractures are treated as outpatients. Diagnostic and procedural codes were sourced from inpatient (Discharge Abstract Database), outpatient (Ontario Health Insurance Plan [OHIP]) and emergency department (National Ambulatory Care Reporting System) data.

Fracture definitions contained diagnostic codes only, or both diagnostic and procedural codes, Appendix A, Table A1. Hip fracture codes were sourced from inpatient and emergency department claims data; there is no diagnostic code for hip fracture in outpatient claims in Ontario [10]. Codes used to identify hip fractures included diagnostic (ICD-10-CA: S72.0x, S72.1x, S72.2x) [11] and procedural codes for hip fixation (Canadian classification of health interventions [CCI]: 1VA74, 1VC74), reduction (CCI: 1VA73, 1VC73), repair (CCI: 1VA80, 1VC80) and replacement (CCI: 1VA53) [11,12]. Deaths were identified from the Registered Persons Database, which contains demographic data on all residents in Ontario who have a health card, including the date of death. To illustrate the interpretation of the definitions listed in Appendix A, Table A1, definition 1 for hip fractures required at least one diagnosis code of hip fracture from inpatient or emergency department data. In contrast, definition 4 required at least one hip fracture diagnosis from inpatient or emergency data with a procedure or death that occurred within 7 days or during the same hospitalization as the initial diagnosis.

Definitions for radius/ulna fractures utilized claims from inpatient, emergency and outpatient data. Codes used to identify radius/ulna fractures included diagnostic (ICD-10-CA: S52.x, OHIP code: 813) [10,11] and procedural codes for fixation (CCI: 1TV74), reduction (CCI: 1TV73), immobilization (CCI: 1TV03), and repair (CCI: 1TV80, 1TV82) [12].

Trauma codes were chosen based on the recommended core ICD-10-CA codes for injury indicators [11,13]. These codes included the “V” and “W” trauma codes. A full list of trauma codes is provided in Appendix A, Table A2.

### 2.3. Statistical Analyses

In our primary analysis, the most inclusive definition (Appendix A, Table A1, definition 1) served as the referent group for comparisons of each fracture site. We identified the first fracture for each patient during follow-up. Our primary outcome was fracture incidence censored on death or end of follow-up (primary cohort: 2020/03; secondary cohort: 2015/03). Hip fracture definitions 11, 12, and 13 were not tested in the secondary cohort because procedures in emergency data were not available for this cohort. We estimated and compared the number of fractures, incidence rates, the proportion of fractures captured (relative to definition 1), and median time to fracture using each fracture definition.

### 2.4. Sensitivity Analyses

We conducted two sensitivity analyses unique to hip fracture: (1) exclusion of the subtrochanteric femoral fracture (ICD-10: S72.2x) diagnosis, and (2) removal of the hip replacement (CCI: 1VA53) procedural code for definitions that required procedural codes. In our previous review [4], we found that diagnosis codes for subtrochanteric femoral fractures (ICD-10: S72.2x) are commonly included in hip fracture definitions associated with osteoporosis. However, atypical femoral fractures (AFF) that occur in the subtrochanteric or shaft regions of the femur have emerged as a rare, yet important adverse event associated with long-term bisphosphonate exposure. Importantly, AFFs are defined by more than anatomical location, and require at least four major radiographic features to be present [14]. Therefore, it was important to include subtrochanteric femoral fractures in our primary analysis. However, we appreciate that fractures coded as subtrochanteric could include some AFFs, and thus we excluded subtrochanteric fractures in a sensitivity analysis. In our second sensitivity analysis specific to hip fractures, hip replacement was excluded from definitions that included procedures because it was unclear how often hip replacement codes were included in application [4]. Yet, procedural codes for hip replacement were used in validation articles for hip fracture [15,16,17]. We also explored the frequency in hip replacement procedures documented with hip fractures identified over time in our cohorts.

In a third sensitivity analysis applied to both fracture sites, we excluded fractures that were associated with trauma codes in an inpatient or emergency department setting [13]. Fourth, we employed a washout window of 180 days between current and prior fractures (fractures identified before the index date) [4]. A washout window of 180 days was chosen based on our previous scoping review as 180 days was the most common period used in previous studies. For these sensitivity analyses, we calculated the proportion and change in the incidence of fracture relative to the primary analysis for each definition.

## 3. Results

### 3.1. Cohorts

Of 120,368 long-term oral bisphosphonate users identified between November 2000 and December 2016, 120,363 had long-term use after April 2003 and were included in our primary cohort. Among these patients, 80% (*n* = 96,032) were female and had a median age of 74.0 years, Appendix A, Table A3. Two-thirds (*n* = 81,046, 67%) lived in major urban areas, and most patients (79%) received their initial oral bisphosphonate prescription from a general practitioner. Of 203,358 older adults exposed to chronic glucocorticoids between January 1998 and September 2014, 140,979 were community-dwelling and exposed after April 2003 and thus included in our secondary cohort. In this cohort, 56% were female, with a median age of 74.0 years.

### 3.2. Primary Analysis

#### 3.2.1. Hip Fractures

In our primary cohort, we identified 52 to 8058 hip fractures with incidence rates from 0.7 to 111.8 per 10,000 person-years between definitions, Table 1. The most inclusive definition only required a single diagnosis from inpatient or emergency data. Definition 2 required one inpatient diagnosis and had an 8% drop in the number of fractures identified relative to definition 1. Of interest, only 94% of the fractures identified by definition 2 were identified if restricted to primary diagnosis only. Definition 3, which only required one diagnosis from emergency data had a 9% drop in the number of fractures identified, relative to definition 1.

Definitions requiring combinations of diagnosis codes and procedures, or death were more restrictive. For example, definition 10, which only used inpatient data and required a procedure or death within 7 days of diagnosis, identified 15% fewer fractures relative to definition 1. Using emergency department data alone and requiring a procedure or death identified the fewest number of fractures, ranging from as few as 4% (definition 11) to <1% (definition 13) of fractures identified relative to definition 1. Median times to fracture ranged from 4.1 to 5.9 years.

#### 3.2.2. Radius/Ulna Fractures

Radius/ulna fractures ranged from 1589 to 6797 with fracture incidence rates from 22.0 to 94.3 per 10,000 person-years, Table 2. Definition 1 was the most inclusive, requiring one diagnosis from inpatient, emergency, or outpatient data. Definition 2 excluded emergency data and identified 9% fewer fractures than the most inclusive definition. Definition 3 required two outpatient diagnoses or one inpatient diagnosis and identified 17% fewer fractures than the most inclusive definition. Outpatient data were important in identifying radius/ulna fractures. Definition 5, one diagnosis from inpatient or emergency data, identified only 78% of the fractures relative to the most inclusive definition. Using inpatient data alone identified the fewest number of fractures, only 23% of fractures relative to the most inclusive definition were identified. There was little variation in median times to fracture, which ranged from 3.6 to 3.8 years.

### 3.3. Sensitivity Analyses

#### 3.3.1. Exclusion of Fractures Associated with Trauma Codes

Excluding fractures associated with trauma had minimal impact on the proportion of hip and radius/ulna fractures captured, with at least 98% (hip) and 95% (radius/ulna) of fractures captured, Table 1 and Table 2.

#### 3.3.2. 180-Day Washout Windows

Washout windows had a minimal effect on the proportion of hip and radius/ulna fractures captured, with 98% of hip fractures and 99% of radius/ulna fractures captured relative to the primary analysis, Table 1 and Table 2.

#### 3.3.3. Exclusion of the S72.2x (Subtrochanteric Femoral Fracture) Diagnosis Code—Hip Fractures

Most definitions had at least 93% of their fractures captured relative to the primary analysis after excluding the diagnosis code for subtrochanteric hip fracture. However, excluding the subtrochanteric femoral fracture diagnosis code had a substantial impact on two definitions that identified fractures from emergency department data (definition 12: 15% decrease in the number of fractures captured; definition 13: 22% decrease in the number of fractures captured), Table 1. Nonetheless, these definitions also identified the lowest number of fractures overall in our primary analysis.

#### 3.3.4. Exclusion of the 1VA53 (Hip Replacement) Procedural Code—Hip Fractures

Excluding the procedural code for hip replacement identified only two-thirds of fractures identified in hospital, yet had little impact on the few identified exclusively using emergency department data, Table 1. Of interest, our sub-analysis that considered use of hip replacement procedures over time found that the rate of hip replacements remained stable (results not shown).

### 3.4. Secondary Cohort

Overall, results were similar in our secondary cohort of patients receiving long-term glucocorticoids, Appendix A, Table A4 and Table A5.

## 4. Discussion

We identified wide variation in the number and rate of fractures using common definitions for hip and radius/ulna fractures and replicated our findings in a second cohort. Our previous review found that the most common definition used to define hip fracture was a single inpatient diagnosis code [4]. In the present study, this definition identified 8% fewer fractures relative to the most inclusive definition that also included emergency department data. Restricting hip fracture identification to inpatient claims is thus a major concern as the number of fractures captured across studies is potentially missing up to 8% of hip fractures that occur.

Real-world evidence is not only used to evaluate safety and effectiveness of medications affecting fracture risk, but is important in identifying fracture trends and projections, risk factors for fracture, and fracture risk in defining treatment thresholds [18,19]. In turn, real-world evidence can influence clinical practice guidelines and impact which patients may receive treatment for osteoporosis. For example, risk factors for fracture are incorporated into treatment guidelines and fracture risk assessments [20]. However, since many hip fracture prediction estimates have been restricted to inpatient data, some risk factors may be underestimated and thus some patients may go untreated.

Another major finding is that definitions that exclude hip replacements missed one-third of the fractures identified in our primary analysis. We conducted this sensitivity analysis because our previous review of osteoporosis drug effects studies that leveraged claims data identified that 40% used procedural codes to define hip fractures [4], yet no study that disclosed their codes listed hip replacement as an eligible procedure, despite hip replacements being used in validation articles [15,16,17]. While it is true that hip replacements may be more common in patients with osteoarthritis rather than fracture, depending on the quality of the bone, a hip replacement may be performed to repair hip fractures [21]. However, we also acknowledge the possibility of ‘code creep’ or ‘upcoding’, a phenomenon in which physicians bill for more expensive procedures over time, as this has previously been documented in outpatient clinics in Ontario [22]. However, code creep has not been documented in Ontario hospitals [23]. Indeed, the rates of hip replacements in our study remained constant over our study period. Thus, our results point to the importance of including hip replacement procedures if procedural codes are included to define hip fracture occurrence.

We also acknowledge that prior studies that required procedural codes to define hip fracture may have included hip replacement as an eligible procedure but did not include a list of procedures or codes, which further highlights the importance of having transparency in describing outcome definitions. Additionally, commonly cited validation articles included hip replacement procedural codes, and thus the estimates of validity depend on the presence of these codes [15,16,17]. Ray et al.’s validation study is the most well-cited and used ICD-9 Medicare claims data from 1987 [15]. Although ICD-9 codes are still used in the United States, they are regularly updated. The hip replacement codes used by Ray et al. (81.61 and 81.62) no longer exist—these were replaced in 1989 [15,24,25]. Currently, the ICD-9 hip replacement codes are 81.51 (total hip replacement), and 81.52 (partial hip replacement) [26]. Indeed, these ICD-9 procedural codes were used in a more recent validation study [16]. Thus, we recommend that study investigators take extra care when selecting codes for identifying fractures and referring to validation articles to ensure that the codes used are current for their study period. Presumably, authors would update codes to include comparable definitions in their data, yet transparency in reporting specific definitions is essential to confirm. As a whole, requiring procedures identified about 10% fewer hip fractures than the most inclusive definition. Although fewer fractures are identified, the definitions requiring procedures or death may be more specific (i.e., fewer false positives) than those requiring a diagnosis alone. In turn, definitions that only require a diagnosis have higher sensitivity (i.e., fewer false negatives). As such, the decision of whether to require a procedure in fracture definitions depends on the needs of the study.

We recommend that the most inclusive fracture definitions be used as primary definitions in studies to ensure that all fractures are captured and fractures from more fragile patients, who may not be able to undergo a procedure, are not missed. More specific definitions that require procedural codes or use washout windows may be used in sensitivity analyses.

Our study has some limitations to note. First, trauma codes are not available in outpatient data and are inconsistently documented in claims data [27]. This is a potential limitation to our sensitivity analysis where we excluded fractures associated with trauma codes for radius/ulna fractures that were more commonly identified in an outpatient setting. However, patients who experience fractures due to trauma would most likely be treated in an emergency or inpatient setting, since the trauma codes we used indicate injuries sustained in traffic accidents and due to serious falls (e.g., fall from scaffolding). Thus, we anticipate misclassification of trauma codes had little impact on our analysis. Second, procedure codes were not available in emergency data for our secondary cohort of patients exposed to chronic glucocorticoids. Although there is potential to miss some fractures due to the lack of procedure codes, most hip fracture procedures would be performed in an inpatient setting and most radius/ulna fracture procedures would occur in an outpatient setting. Thus, the number of procedures missed would be few. Lastly, our study did not validate our fracture definitions with chart review. However, Ontario healthcare administrative data have high validity for fractures in hospital, with 95% sensitivity and 95% positive predictive value for hip fracture diagnoses (ICD-10 code: S72.x), and high validity overall for diagnoses in emergency department data (86% agreement between claims data and medical charts) [28,29,30].

## 5. Conclusions

In this study, we showed wide variation in the number of fractures identified by different hip and radius/ulna fracture definitions, which has an impact on fracture incidence estimates. We recommend that the most inclusive fracture definitions be used as primary definitions in studies. Further research investigating the impact of fracture identification using health claims data on risk factor, drug safety, and drug effectiveness estimates is warranted to inform best practice in fracture outcome identification.

## Figures and Tables

**Table 1:** Hip fractures identified, N = 120,363.

	Primary Analysis				Sensitivity Analyses							
					Exclude Subtrochanteric Fractures		Exclude Hip Replacement		Exclude Fractures Associated with Trauma		180-Day Washout	
Definition	Fractures	Captured	Incidence	Median Time to Fracture	% of Primary	Δ Incidence	% of Primary	Δ Incidence	% of Primary	Δ Incidence	% of Primary	Δ Incidence
Number, Description	(N)	(%)	10,000 PYs	Years [IQR]	(%)	10,000 PYs	(%)	10,000 PYs	(%)	10,000 PYs	(%)	10,000 PYs
1 *: IE, dx	8058	100.0	111.8	4.1 [1.9–6.8]	95.4	5.1	--	--	98.8	1.3	99.9	0.1
2: I, dx ^±^	7447	92.4	103.3	4.1 [1.9–6.8]	93.2	7.0	--	--	98.4	1.6	99.9	0.0
3: E, dx	7315	90.8	101.5	4.1 [1.9–6.8]	95.6	4.5	--	--	98.4	1.6	100.0	0.0
4: IE, dx + (px/death) in 7 days or **	7173	89.0	99.5	4.1 [1.9–6.8]	95.0	5.0	68.8	31.1	98.4	1.9	99.9	0.0
5: IE, dx + (px/death) **	7044	87.4	97.7	4.1 [1.9–6.8]	95.0	4.8	67.9	31.3	98.4	1.6	99.9	0.0
6: I, dx + (px/death) **	7006	86.9	97.2	4.2 [1.9–6.8]	92.7	7.1	69.4	29.7	98.4	1.5	100.0	0.0
7: IE, dx + px within 7 days	6915	85.8	95.9	4.2 [1.9–6.8]	95.0	4.8	66.1	32.5	98.3	1.5	99.9	0.0
8: IE, dx + px **	6889	85.5	95.6	4.2 [1.9–6.8]	92.8	6.9	65.9	32.6	98.3	4.3	100.0	0.1
9: I, dx + (px/death) in 7 days	6903	85.7	95.8	4.2 [1.9–6.8]	92.9	6.9	67.6	31.1	98.4	1.6	100.0	0.0
10: I, dx + px within 7 days	6797	84.4	94.3	4.2 [1.9–6.8]	92.8	6.8	65.9	32.1	98.4	1.5	100.0	0.0
11: E, dx + (px=/death) in 7 days	316	3.9	4.4	4.0 [1.9–7.0]	93.4	0.3	100.0	0.0	99.1	0.1	100.0	0.0
12: E, dx + px	79	1.0	1.1	4.5 [1.7–7.6]	84.8	0.2	100.0	0.0	98.7	0.0	100.0	0.0
13: E, dx + px in 7 days	52	0.6	0.7	5.9 [3.0–8.4]	78.8	0.1	100.0	0.0	98.1	0.0	100.0	0.0

dx = diagnosis code; E = emergency department data; I = inpatient data; IQR = Interquartile range; PYs = person-years; px = procedure code; Δ = absolute change in incidence relative to primary; * primary (referent) definition; ^±^ restricting this definition to primary diagnosis identified 6990 fractures; ** in same hospitalization.

**Table 2:** Radius/ulna fractures identified, N = 120,363.

	Primary Analysis				Sensitivity Analyses			
					Exclude Fractures Associated with Trauma		180-Day Washout	
Definition	Fractures	Captured	Incidence	Median Time to Fracture	% of Primary	Δ Incidence	% of Primary	Δ Incidence
Number, Description	(N)	(%)	Per 10,000 PYs	Years [IQR]	(%)	Per 10,000 PYs	(%)	Per 10,000 PYs
1 *: IEO, dx	6797	100.0	94.3	3.6 [1.5–6.3]	99.5	0.4	99.0	0.9
2: IO, dx	6193	91.1	85.9	3.6 [1.5–6.3]	99.7	0.2	99.7	0.3
3: IO, dx ^±^	5659	83.3	8.5	3.6 [1.6–6.3]	100.0	0.0	99.7	0.2
4: IEO, dx + px in 7 days	5355	78.8	4.3	3.7 [1.6–6.5]	99.6	0.3	99.7	0.2
5: IE, dx	5310	78.1	3.7	3.8 [1.7–6.6]	97.2	2.1	99.8	0.2
6: dx + px within 90 days	4850	71.4	67.3	3.7 [1.6–6.5]	100.0	0.0	100.0	0.0
7: I, dx	1589	23.4	22.0	3.6 [1.7–6.7]	95.4	1.0	99.9	0.0

dx = diagnosis code; E = emergency department data; I = inpatient data; IQR = Interquartile range; O = outpatient data; PYs = person-years; px = procedure code; Δ = absolute change in incidence relative to primary; * primary (referent) definition; ^±^ two diagnoses required for outpatient data within 90 days of each other.

## Data Availability

The underlying analytic code is available from the authors upon request, understanding that the computer programs may rely upon coding templates or macros that are unique to ICES and are therefore either inaccessible or may require modification.

## Appendix A

**Table A1:** Fracture definitions identified in the literature.

Data Source(s)	Definition				Codes Occurring within Below Timeframe		
	#	D	Px	Px or Death	7 Days	Same Hospitalization	90 Days
Hip Fractures							
Inpatient, Emergency	1	X					
Inpatient	2	X					
Emergency	3	X					
Inpatient, Emergency	4	X		X	X	X	
	5	X		X	X		
Impatient	6	X		X		X	
Impatient, Emergency	7	X	X		X		
Inpatient	8	X	X			X	
	9	X		X	X		
	10	X	X		X		
Emergency	11	X		X	X		
	12	X	X				
	13	X	X		X		
**Radius/Ulna Fractures**							
Inpatient, Emergency, Outpatient	1	X					
Inpatient, Outpatient	2	X					
	3	X ^a^					X ^b^
Inpatient, Emergency, Outpatient	4	X	X		X		
Inpatient, Emergency	5	X					
Outpatient	6	X	X				X
Inpatient	7	X					

Dx—diagnosis; Px—procedure; ^a^ two dx required for outpatient; ^b^ outpatient only.

**Table A2:** Trauma codes and descriptions.

Code	Description
V01	Pedestrian injured in collision with pedal cycle
V02	Pedestrian injured in collision with two- or three-wheeled motor vehicle
V03	Pedestrian injured in collision with car, pick-up truck or van
V04	Pedestrian injured in collision with heavy transport vehicle or bus
V05	Pedestrian injured in collision with railway train or railway vehicle
V06	Pedestrian injured in collision with other nonmotor vehicle
V09	Pedestrian injured in other and unspecified transport accidents
V10	Pedal cyclist injured in collision with pedestrian or animal
V11	Pedal cyclist injured in collision with other pedal cycle
V12	Pedal cyclist injured in collision with two- or three-wheeled motor vehicle
V13	Pedal cyclist injured in collision with car, pick-up truck or van
V14	Pedal cyclist injured in collision with heavy transport vehicle or bus
V15	Pedal cyclist injured in collision with railway train or railway vehicle
V16	Pedal cyclist injured in collision with other nonmotor vehicle
V17	Pedal cyclist injured in collision with fixed or stationary object
V18	Pedal cyclist injured in noncollision transport accident
V19	Pedal cyclist injured in other and unspecified transport accidents
V20	Motorcycle rider injured in collision with pedestrian or animal
V21	Motorcycle rider injured in collision with pedal cycle
V22	Motorcycle rider injured in collision with two- or three-wheeled motor vehicle
V23	Motorcycle rider injured in collision with car, pick-up truck or van
V24	Motorcycle rider injured in collision with heavy transport vehicle or bus
V25	Motorcycle rider injured in collision with railway train or railway vehicle
V26	Motorcycle rider injured in collision with other nonmotor vehicle
V27	Motorcycle rider injured in collision with fixed or stationary object
V28	Motorcycle rider injured in noncollision transport accident
V29	Motorcycle rider injured in other and unspecified transport accidents
V30	Occupant of three-wheeled motor vehicle injured in collision with pedestrian or animal
V31	Occupant of three-wheeled motor vehicle injured in collision with pedal cycle
V32	Occupant of three-wheeled motor vehicle injured in collision with two- or three-wheeled motor vehicle
V33	Occupant of three-wheeled motor vehicle injured in collision with car, pick-up truck or van
V34	Occupant of three-wheeled motor vehicle injured in collision with heavy transport vehicle or bus
V35	Occupant of three-wheeled motor vehicle injured in collision with railway train or railway vehicle
V36	Occupant of three-wheeled motor vehicle injured in collision with other nonmotor vehicle
V37	Occupant of three-wheeled motor vehicle injured in collision with fixed or stationary object
V38	Occupant of three-wheeled motor vehicle injured in noncollision transport accident
V39	Occupant of three-wheeled motor vehicle injured in other and unspecified transport accidents
V40	Car occupant injured in collision with pedestrian or animal
V41	Car occupant injured in collision with pedal cycle
V42	Car occupant injured in collision with two- or three-wheeled motor vehicle
V43	Car occupant injured in collision with car, pick-up truck or van
V44	Car occupant injured in collision with heavy transport vehicle or bus
V45	Car occupant injured in collision with railway train or railway vehicle
V46	Car occupant injured in collision with other nonmotor vehicle
V47	Car occupant injured in collision with fixed or stationary object
V48	Car occupant injured in noncollision transport accident
V49	Car occupant injured in other and unspecified transport accidents
V50	Occupant of pick-up truck or van injured in collision with pedestrian or animal
V51	Occupant of pick-up truck or van injured in collision with pedal cycle
V52	Occupant of pick-up truck or van injured in collision with two- or three-wheeled motor vehicle
V53	Occupant of pick-up truck or van injured in collision with car, pick-up truck or van
V54	Occupant of pick-up truck or van injured in collision with heavy transport vehicle or bus
V55	Occupant of pick-up truck or van injured in collision with railway train or railway vehicle
V56	Occupant of pick-up truck or van injured in collision with other nonmotor vehicle
V57	Occupant of pick-up truck or van injured in collision with fixed or stationary object
V58	Occupant of pick-up truck or van injured in noncollision transport accident
V59	Occupant of pick-up truck or van injured in other and unspecified transport accidents
V60	Occupant of heavy transport vehicle injured in collision with pedestrian or animal
V61	Occupant of heavy transport vehicle injured in collision with pedal cycle
V62	Occupant of heavy transport vehicle injured in collision with two- or three-wheeled motor vehicle
V63	Occupant of heavy transport vehicle injured in collision with car, pick-up truck or van
V64	Occupant of heavy transport vehicle injured in collision with heavy transport vehicle or bus
V65	Occupant of heavy transport vehicle injured in collision with railway train or railway vehicle
V66	Occupant of heavy transport vehicle injured in collision with other nonmotor vehicle
V67	Occupant of heavy transport vehicle injured in collision with fixed or stationary object
V68	Occupant of heavy transport vehicle injured in noncollision transport accident
V69	Occupant of heavy transport vehicle injured in other and unspecified transport accidents
V70	Bus occupant injured in collision with pedestrian or animal
V71	Bus occupant injured in collision with pedal cycle
V72	Bus occupant injured in collision with two- or three-wheeled motor vehicle
V73	Bus occupant injured in collision with car, pick-up truck or van
V74	Bus occupant injured in collision with heavy transport vehicle or bus
V75	Bus occupant injured in collision with railway train or railway vehicle
V76	Bus occupant injured in collision with other nonmotor vehicle
V77	Bus occupant injured in collision with fixed or stationary object
V78	Bus occupant injured in noncollision transport accident
V79	Bus occupant injured in other and unspecified transport accidents
V80	Animal-rider or occupant of animal-drawn vehicle injured in transport accident
V81	Occupant of railway train or railway vehicle injured in transport accident
V82	Occupant of streetcar injured in transport accident
V83	Occupant of special vehicle mainly used on industrial premises injured in transport accident
V84	Occupant of special vehicle mainly used in agriculture injured in transport accident
V85	Occupant of special construction vehicle injured in transport accident
V86	Occupant of special all-terrain or other motor vehicle designed primarily for off-road use, injured
V87	Traffic accident of specified type but victim’s mode of transport unknown
V88	Nontraffic accident of specified type but victim’s mode of transport unknown
V89	Motor- or nonmotor-vehicle accident, type of vehicle unspecified
V90	Accident to watercraft causing drowning and submersion
V91	Accident to watercraft causing other injury
V92	Water-transport-related drowning and submersion without accident to watercraft
V93	Accident on board watercraft without accident to watercraft, not causing drowning and submersion
V94	Other and unspecified water transport accidents
V95	Accident to powered aircraft causing injury to occupant
V96	Accident to nonpowered aircraft causing injury to occupant
V97	Other specified air transport accidents
V98	Other specified transport accidents
V99	Unspecified transport accidents
W03	Other fall on same level due to collision with, or pushing by, another person
W04	Fall while being carried or supported by other persons
W09	Fall involving playground equipment
W11	Fall on and from ladder
W12	Fall on and from scaffolding
W13	Fall from, out of or through building or structure
W14	Fall from tree
W15	Fall from cliff
W16	Diving or jumping into water causing injury other than drowning or submersion

**Table A3:** Characteristics of patients in the primary cohort (N = 120,363).

Characteristic	N (%)
Sex, female	96,032 (79.8%)
Age, median [interquartile range]	74.0 [69.0–80.0]
*Rurality Index of Ontario*	
Major Urban	81,046 (67.3%)
Nonmajor urban	20,788 (17.3%)
Rural	4305 (3.6%)
Missing	14,224 (11.8%)
*Comorbidities*	
Asthma	14,174 (11.8%)
Chronic obstructive pulmonary disease	22,500 (18.7%)
Congestive heart failure	8195 (6.8%)
Crohn’s and Colitis	904 (0.8%)
Diabetes	23,533 (19.6%)
Hypertension	80,515 (66.9%)
Myocardial infarction	3675 (3.1%)
Rheumatoid arthritis	4120 (3.4%)
*Prescriber Specialty*	
Family and general practice	94,901 (78.9%)
Geriatrics	1660 (1.4%)
Endocrinology	1110 (0.9%)
Rheumatology	3779 (3.14)
Other	4912 (4.1%)
Missing	14,001 (11.6%)

**Table A4:** Hip fractures identified in the secondary cohort, N = 140,979.

	Primary Analysis				Sensitivity Analyses							
					Exclude Subtrochanteric Fractures		Exclude Hip Replacement		Exclude Fractures Associated with Trauma		180-Day Washout	
Definition	Fractures	Captured	Incidence	Median Time to Fracture	% of Primary	Δ Incidence	% of Primary	Δ Incidence	% of Primary	Δ Incidence	% of Primary	Δ Incidence
Number, Description	(N)	(%)	Per 10,000 PYs	Years [IQR]	(%)	Per 10,000 PYs	(%)	Per 10,000 PYs	(%)	Per 10,000 PYs	(%)	Per 10,000 PYs
1 *: IE, dx	4991	100.00	88.9	2.9 [1.2–5.4]	98.0	1.7	--	--	98.5	1.3	100.0	0.0
2: I, dx	4614	92.4	82.2	2.9 [1.2–5.4]	96.6	2.8	--	--	98.2	1.5	100.0	0.0
3: E, dx	4422	88.6	78.8	2.9 [1.3–5.5]	97.6	1.9	--	--	98.0	1.6	100.0	0.0
4: IE, dx + (px or death) in 7 days or ^×^	4455	89.3	79.4	2.9 [1.2–5.4]	97.9	1.7	64.2	28.4	98.0	1.5	100.0	0.0
5: IE, dx + (px or death) ^×^	4306	86.3	76.7	2.9 [1.3–5.5]	98.0	1.6	61.9	29.2	98.0	1.5	100.0	0.0
6: I, dx + (px or death) ^×^	4332	86.8	77.2	2.9 [1.3–5.5]	96.4	2.8	64.1	27.8	98.0	1.5	100.0	0.0
7: IE, dx + px within 7 days	4187	83.9	74.6	2.9 [1.3–5.5]	97.9	1.5	59.3	30.4	98.0	1.5	100.0	0.0
8: IE, dx + px ^×^	4202	84.2	74.9	2.9 [1.3–5.5]	96.4	2.7	59.3	30.5	98.0	1.5	100.0	0.0
9: I, dx + (px or death) in 7 days	4210	84.4	75.0	2.9 [1.3–5.5]	96.5	2.6	61.7	28.7	98.0	1.5	100.0	0.0
10: I, dx + px within 7 days	4108	82.3	73.2	3.0 [1.3–5.5]	96.5	2.6	59.2	29.9	98.0	1.5	100.0	0.0

* Primary (referent) definition; ^×^ in same hospitalization; Δ = absolute change in incidence relative to primary; IQR = interquartile range, PYs = person-years; I = inpatient; E = emergency department; dx = diagnosis; px = procedure.

**Table A5:** Radius/ulna fractures identified in the secondary cohort, N = 140,979.

	Primary Analysis				Sensitivity Analyses			
					Exclude Fractures Associated with Trauma		180-Day Washout	
Definition	Fractures	Captured	Incidence	Median Time to Fracture	% of Primary	Δ Incidence	% of Primary	Δ Incidence
Number, Description	(N)	(%)	Per 10,000 PYs	Years [IQR]	(%)	Per 10,000 PYs	(%)	Per 10,000 PYs
1 *: IEO, dx	4645	100.00%	82.1	2.7 [1.2–5.1]	99.5	0.2	99.5	0.3
2: IO, dx	4268	91.9%	6.1	2.7 [1.2–5.2]	99.8	0.2	99.8	0.1
3: IO, dx ^±^	3757	80.9%	67.0	2.7 [1.2–5.2]	100.0	0.0	99.8	0.1
4: IEO, dx + px in 7 days	3469	74.7%	61.8	2.8 [1.3–5.4]	99.6	0.3	99.9	0.1
5: IE, dx	3056	65.8%	54.5	2.8 [1.3–5.1]	97.2	1.5	100.0	0.0
6: dx + px within 90 days	3220	69.3%	57.4	2.9 [1.3–5.4]	100.0	0.0	100.0	0.0
7: I, dx	893	19.2%	15.9	3.0 [1.4–5.2]	95.7	0.7	100.0	0.0

* Primary (referent) definition; ^±^ two diagnoses required for outpatient data within 90 days of each other; Δ = absolute change in incidence relative to primary; IQR = interquartile range; PYs = person-years; I = inpatient; E = emergency department; O = outpatient; dx = diagnosis; px = procedure.
